# Tisp40 Induces Tubular Epithelial Cell GSDMD-Mediated Pyroptosis in Renal Ischemia-Reperfusion Injury via NF-κB Signaling

**DOI:** 10.3389/fphys.2020.00906

**Published:** 2020-08-13

**Authors:** Chengcheng Xiao, Haijun Zhao, Hai Zhu, Yingyu Zhang, Qiuju Su, Feng Zhao, Renhe Wang

**Affiliations:** ^1^Department of Urology, Qingdao Municipal Hospital, Qingdao University, Qingdao, China; ^2^Department of Traditional Chinese Medicine, Qingdao Municipal Hospital, Qingdao University, Qingdao, China

**Keywords:** renal ischemia-reperfusion injury, Tisp40, pyroptosis, tubular epithelial cell, NF-κB signaling

## Abstract

Renal ischemia-reperfusion injury (IRI) is a major cause of acute kidney injury (AKI). As a transcription factor, the Transcript induced in spermiogenesis 40 (Tisp40) has been found to be involved in renal IRI. However, the role of Tisp40 in tubular epithelial cell (TEC) pyroptosis of renal IRI remains unknown. In this study, we investigated effects of Tisp40 on Gasdermin D (GSDMD)-mediated TEC pyroptosis in renal IRI and underlying molecular mechanisms in I/R-induced kidney by hematoxylin and eosin (HE) staining, Terminal deoxynucleotidyl transferase dUTP nick-end labeling (TUNEL) assay,immunohistochemistry (IHC), reverse transcription-quantitative PCR (RT-qPCR) and western blot analysis *in vivo* and oxygen-glucose deprivation/reoxygenation (OGD/R)-stimulated TCMK-1 cells by lactate dehydrogenase (LDH) release assay, CCK-8 assay,enzyme-linked immunosorbent assay (ELISA), flow cytometric analysis, immunofluorescence staining,RT-qPCRand western blot analysis *in vitro*. We found that the levels of Tisp40 and GSDMD-N expression increased gradually, and peaked at 30 min ischemia/24 h reperfusion *in vivo* and 24 h OGD/R/6 h reoxygenation *in vitro*, simultaneously, the levels of TEC pyroptosis and renal injury were correspondingly increased. The data of Pearson’s correlation analysis showed that the expression of Tisp40 and GSDMD-N was positively correlated. Furthermore, Tisp40 overexpression aggravated TEC pyroptosis rate and increased the expressions of related proteins, including GSDMD-N, NLRP3, caspase-1, IL-1β, and IL-18 in the OGD/R-stimulated TCMK-1 cell line, whereas the opposite occurred in cells treated with small interfeing RNA (siRNA) targeting Tisp40. Tisp40-deficient mice showed attenuated renal IRI and pyroptosis compared with wild-type mice. In addition, Tisp40 knockout remarkably decreased the levels of GSDMD-N, IL-1β, IL-18, NLRP3, and caspase-1 expression, and alleviated renal pyroptosis induced by I/R. Importantly, Tisp40 overexpression significantly increased TECs pyroptosis via p-p65 activation, however, the effects of Tisp40 overexpression were partially blocked by parthenolide (PTL). Collectively, our findings provide insight into the mechanism of how Tisp40 regulated GSDMD-mediated pyroptosis in renal IRI.

## Introduction

Acute kidney injury (AKI) is a common complication in clinical inpatients and shows a morbidity and mortality of approximately 5% and 50–80%, respectively. (Gill et al., 2005; Perico and Remuzzi, 2015). Approximately 2 million patients die from ischemic AKI every year worldwide (Tao et al., 2013). Continuous inflammatory stimulation of the tubule interstitium significantly increases the risk of chronic kidney disease (CKD) and end-stage renal disease (ESRD) in surviving patients (Chawla et al., 2014). Currently, no effective prevention or cure measures for AKI have been established in clinical practice (Arai et al., 2016). Renal ischemia-reperfusion injury (IRI) is one of the main causes of AKI (Jha et al., 2013) and is secondary to various clinical conditions, including kidney transplantation, cardiopulmonary and aortic aneurysm surgeries, severe hemorrhagic shock, and endotoxin sepsis (Gill et al., 2005; Bastin et al., 2013; Noh et al., 2015). Tubular epithelial cells (TECs) are likely injured by high metabolic activity and hypoxia (Al-Bataineh et al., 2016). Determining the underlying mechanism of TEC injury is critical for better understanding the progression of renal IRI and preventing AKI and its evolution to CKD and ESRD.

As a form of programmed cell death, pyroptosis is characterized by excessive cell death and inflammation (Miao et al., 2010). Gasdermin D (GSDMD) is a key downstream effector in cell pyroptosis (Kovacs and Miao, 2017). It forms pores in the plasma membrane that ultimately cause cell swelling, membrane lysis, and inflammatory cytokines release (Shi et al., 2015). GSDMD-mediated pyroptosis of renal TECs is an essential process in renal ischemia-reperfusion (I/R) injury (Diao et al., 2019; Tajima et al., 2019; Liu et al., 2020). Thus, studies are needed to determine the biological networks involved in GSDMD-mediated pyroptosis in TEC injury induced by renal IR.

The transcript induced in spermiogenesis 40 (Tisp40), also named CREB3L4, AIbZIP, and ATCE1, belongs to the CREB/ATF family (Velpula et al., 2012). As a transcription factor, Tisp40 is involved in initiation of the transcription of multiple genes by binding to various response elements, such as the CRE response element, nuclear factor (NF)-κB response element, and unfolded protein response element (Asada et al., 2011). Tisp40 has been reported to be involved in the pathogenesis of renal IRI, and Tisp40 deficiency inhibits and delays IRI (Qin et al., 2017, 2018; Xiao et al., 2017). In this study, we further investigated the potential molecular mechanism of Tisp40 and whether it is involved in regulating TEC pyroptosis in renal IRI.

## Materials and Methods

### Animals and Surgical Protocols

All surgical procedures were conducted according to the Guide for the Care and Use of Laboratory Animals and approved by the Institutional Animal Care and Use Committee of Qingdao Municipal Hospital. C57BL/6 mice (Male, 8 week-old, 20–25 g) were purchased from Beijing Huafukang bioscience Co., Inc (Beijing, China). Tisp40-knockout mice (RIKEN BioResource Center, Stock NO. RBRC01942) with a C57BL/6 background had been described previously (Xiao et al., 2017). According to random number table, the mice were assigned into four groups: (1) wild-type sham-operated; (2) Tisp40-knockout sham-operated; (3) wild-type renal I/R; and (4) Tisp40-knockout renal I/R (*n* = 6 per group). The mice were intraperitoneally anesthetized with 2% sodium phenobarbital (50 mg/kg). The mice, whose desired depth of surgical anesthesia were judged by loss of righting reflex, were bedded onto a homeothermic table to keep a rectal temperature of 38°C approximately. Bilateral pedicles were exposed entirely, and ischemia was induced by pedicels clamping. After 30 min, the clips were removed, and kidneys were collected after 0, 6, 12, 24, and 48 h of reperfusion. The sham animals were subjected to above operation without pedicels clamping. After experiment, the mice were sacrificed by pentobarbital sodium overdose. The left kidneys were removed, and then snap-frozen in liquid nitrogen and stored at −80°C or fixed in 4% paraformaldehyde for biochemical analysis and pathological evaluation.

### Cell Culture

TCMK-1 cells (no. CCL-139) were purchased from American Type Culture Collection. A total of 1.5 × 10^6^ cells/ml cells for group comparison was cultured in complete medium under a 95% air and 5% CO_2_ condition. Cells in OGD/R groups were stimulated with glucose-free medium under hypoxic conditions for 24 h. Then the cells were cultured in complete medium under normal conditions for 0, 2, 4, 6, and 8 h. To knockdown Tisp40, cells were intervened with siRNA-Tisp40 (100 nM, forward, 5′-AGUAGAAGUUGGCCACUUCCAUGGG-3′, reverse, 5′-CCCAUGGAAGUGGCCAACUUCUACU-3′), whose inhibitory effect had been confirmed previously (Xiao et al., 2017) or a scrambled siRNA (forward, 5′-CCUACGCCACCAAUUUCGUdTdT-3′, reverse, 5′-ACGAAAU UGGUGGCGUAGGdTdT-3′) for 12 h using Lipofectamine RNA iMAX (Invitrogen).

### Establishment of Tisp40-Overexpression Cell Line

TCMK-1/Tisp40 (Tisp40-overexpression group) and TCMK-1/vector (empty vector group) cells were obtained as described previously (Xiao et al., 2017). In brief, pUC57-Tisp40 and pLVX-mCMV-ZsGreen-IRES-Puro (Wuhan Viral therapy Technologies Co., Ltd.) were digested by EcoRI and SpeI (NEB), and the products were connected by T4 DNA Ligase (BM121, Transgen BioTech). Then, an Agarose Gel Extraction Kit (D2500, Omega) was used to retrieve fragments. JM109 (Wuhan Viral therapy Technologies Co., Ltd.), which was transfected with the ligation product, was inoculated and amplificated, and the sequencing primer CMV-F, whose primer sequence is 5′-CGCAAATGGGCGGTAGGCGTG-3′, was used to verify the bacterial fluid. rLV-Tisp40 and rLV-ShRNA2 containing the target gene was obtained in 293T cells co-transfected with recombinant plasmid or control plasmid using a Lentiviral Packaging Kit (R003, Wuhan Viral therapy Technologies Co., Ltd.). TCMK-1 cells were transfected with rLV-Tisp40 and rLV-ShRNA2 on the basis of MOI = 20. Two days after transfection, lentivirus-infected TCMK-1 cells were cultured normally.

### HE Staining

Four percent paraformaldehyde was used to fix kidney tissues. The fixed tissues were paraffin embedded and cut into 5-μm-thick sections. Sections were stained using hematoxylin and eosin (HE Kit; Beyotime), according to Kit instructions. Five images were randomly selected under a microscope (×400) to analyze pathological changes. Tubule injury scores were referred to the percentage of tubules affected as follows: 0 ≤ 10%, 1 = 10–25%, 2 = 26–50%, 3 = 51–75%, and 4 ≥ 75%.

### Terminal Deoxynucleotidyl Transferase dUTP Nick-End Labeling (TUNEL) Assay

A total of 5 μm thick sections were stained using a TUNEL kit (Roche). The brown stained cell nuclei were identified as Tunel-positive. Five images were randomly selected under a microscope (×400) to analyze cell apoptosis level. The numbers of the TUNEL-positive TECs in the five fields of renal cortex region for each section were counted at ×400 magnification.

### CCK-8 Assay

Cell viability was measured using a CCK-8 assay kit (C0038, Beyotime) according to the manufacturer’s instructions. The absorbance at 450 nm was measured using a microplate reader (Molecular Devices).

### Lactate Dehydrogenase (LDH) Release Assay

The cells were inoculated into 96-well plates and the culture supernatants were collected. Then, the levels of LDH release were examined using a LDH release assay kit (Beyotime) following the instructions. The absorbance at 495 nm was measured using a microplate reader.

### Reverse Transcription-Quantitative PCR (RT-qPCR)

Total RNA from renal samples and TCMK-1 cells were isolated with TRIzol reagent (Invitrogen). Then Revert Aid First Strand cDNA Synthesis kit (cat. no. K1622; Invitrogen) was used to quantify total RNA and reverse transcribe into cDNA, according to the manufacturer’s instructions. The real-time quantitative PCR for β-actin, Tisp40, NLRP3 and caspase-1 were carried out using the following primers to amplify genes:Tisp40 forward, 5′- TACCTGAAGCCCCAACTACAAA -3′ and reverse, 5′- GTGCCCTGCCACATGATAAA -3′; NLRP3 forward, 5′- ATGCTGCTTCGACATCTCCT -3′ and reverse, 5′- AACCAATGCGAGATCCTGAC -3′; caspase-1 forward, 5′- CACAGCTCTGGAGATGGTGA -3′ and reverse, 5′- TCTTTCAAGCTTGGGCACTT -3′; β-actin forward, 5′- CTGAGAGGGAAATCGTGCGT-3′ and reverse, 5′- CCACAGGATTCCATACCCAAGA-3′. The relative mRNA expression levels were determined using the 2-ΔΔCq method (Livak and Schmittgen, 2001).

### Enzyme-Linked Immunosorbent Assay (ELISA)

The levels of Interleukin (IL)-1β and IL-18 expression in the medium were detected using IL-1β and IL-18 ELISA kits (IL-1β, cat. no.ab197742; IL-18, cat. no.ab216165, Abcam), according to the manufacturer’s instructions. The absorbance at 495 nm was measured using a microplate reader, and ELISA signal was read with a plate reader.

### Flow Cytometric Analysis of Apoptosis

After treatment, the cells were stained with Annexin V, followed by an additional stain with propidium iodide using the Annexin V-FITC Apoptosis Detection kit (BD Biosciences). Subsequently, apoptotic cells were analyzed by flow cytometry (BD Biosciences).

### Immunohistochemistry (IHC)

After deparaffinized and hydrated, the 5-μm-thick sections were blocked with 3% hydrogen peroxide. Then sections were treated with 10% normal goat serum (cat. no. 16210072; Gibco; Thermo Fisher Scientific, Inc.), and incubated overnight following antibodies targeting pro-IL-1β(1:100; cat no. ab205924), IL-1β(1:100;cat no. ab9722) and IL-18 (1:200; cat. no. ab71495). After that, sections were incubated with horseradish peroxidase (HRP)-conjugated goat anti-rabbit secondary antibodies (1:400; cat. no. A32731; Invitrogen). The slides were subsequently stained with DAB to develop the color. The images (magnification, ×400) were captured with a microscope. The percentage of area stained represents the ratio of the summed absolute areas of staining versus the total tissue.

### Immunofluorescence Staining

Four percent paraformaldehyde were used to fix TCMK-1 cells, afterward which were permeabilized with 0.1% Triton X-100, followed by blocked in 10% donkey serum (Sigma-Aldrich). Next, the cells were incubated using following antibodies against GSDMD-N (1:200bs-14287R, Bioss) overnight. The cells were incubated with a secondary antibody (1:400; cat. no. A32731; Invitrogen). DAPI (Roche) was used to nuclei staining. The images (magnification, ×400) were captured with a fluorescent microscope (Leica).

### Western Blotting

Proteins from kidney tissues or cultured TCMK-1 cells were extracted using a Nuclear-Cytosol Extraction Kit (KGP150, Nanjing KeyGen Biotech. Co., Ltd.). Total protein levels were quantified with a BCA assay kit (Invitrogen). The expressions of Tisp40, GSDMD-N, NLRP3, Caspase-1, p65,p-p65 and β-actin was measured. The membranes were incubated overnight with the following antibodies: anti-Tisp40 (1: 100; sc-54800; Santa), anti-GSDMD-N (1:100,bs-14287R, Bioss), anti-NLRP3 (1:200; cat. no. ab214185, Abcam), anti-Caspase-1 (1:200; cat. no. ab138483, Abcam), anti-p65 (1:200; cat. no. ab32536, Abcam), anti-p-p65 (1:200; cat. no. ab86299, Abcam) and β-actin (1:500; cat. no. ab8224, Abcam). Subsequently membranes were incubated with HRP-conjugated secondary antibodies (1:500; cat. no. ab6789, Abcam). Protein bands were visualized and detected using Odyssey Infrared Imaging system Model 9120 (LI-COR Biotechnology).

### Statistical Analysis

Statistical analysis was conducted using the SPSS version 22.0 software, and all data were presented as the mean ± standard deviation (SD). Linear correlation between variables was tested by pearson’s correlation. All experiments were performed ≥3 times. Analysis of variance was used to evaluate statistical significance. *P*-values <0.05 was considered to show a statistically significant difference.

## Results

### Tisp40 Was Closely Related to in GSDMD-Mediated Pyroptosis in I/R-Induced Kidney and OGD/R-Stimulated TECs

**FIGURE 1:**
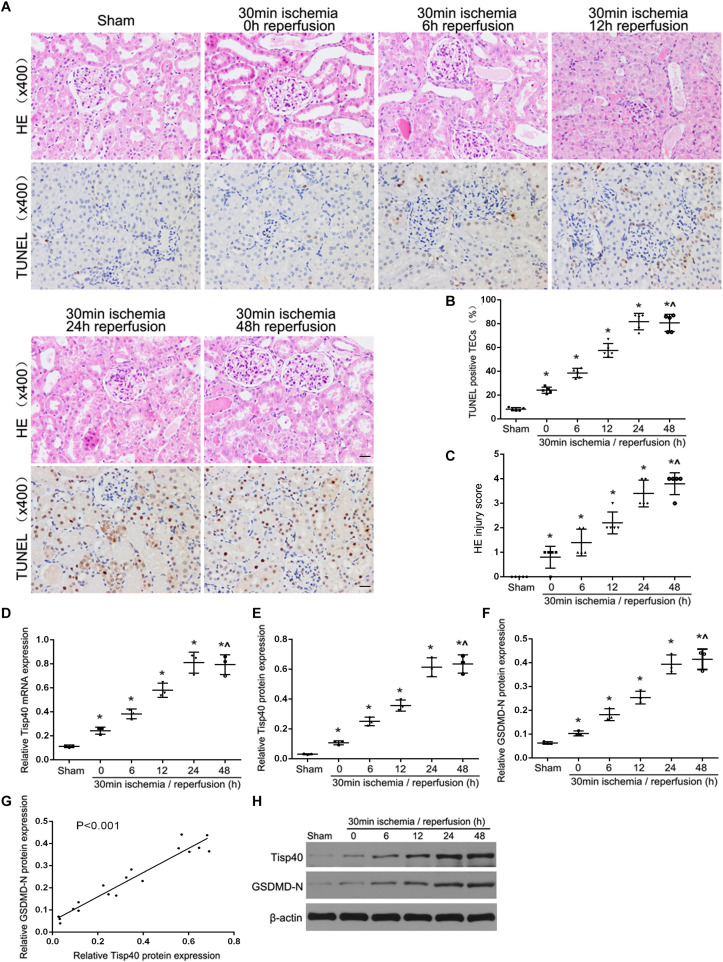
Tisp40 and GSDMD-mediated pyroptosis of TECs were induced in I/R-induced kidney. **(A)** Representative photomicrographs of tubular cell injury in mouse kidney tissue sections with HE and TUNEL staining, 400×, scale bar = 20 μm. **(B)** Statistical analysis showed the percentage of TUNEL positive TECs in the kidney tissues exposed to 30 min ischemia followed by reperfusion of different durations. **(C)** Statistical quantification analysis showed the injury score of HE staining in the kidney tissues exposed to 30 min ischemia followed by reperfusion of different durations. **(D,E,H)** qRT-PCR and western blot analysis of Tisp40 expression at different reperfusion times after 30 min ischemia in kidney samples. **(F,H)** Western blot analysis of GSDMD-N protein expression at different reperfusion times after 30 min ischemia in kidney samples. **(G)** The correlation between the protein expression of Tisp40 and GSDMD-N was analyzed by Pearson’s correlation analysis in the kidney tissues. Data are expressed as the mean ± SD. *n* = 6 per group. *P < 0.05 vs. sham, ^∧^*P* > 0.05 vs. 30 min ischemia and reperfusion of 24 h, one-way ANOVA.

**FIGURE 2:**
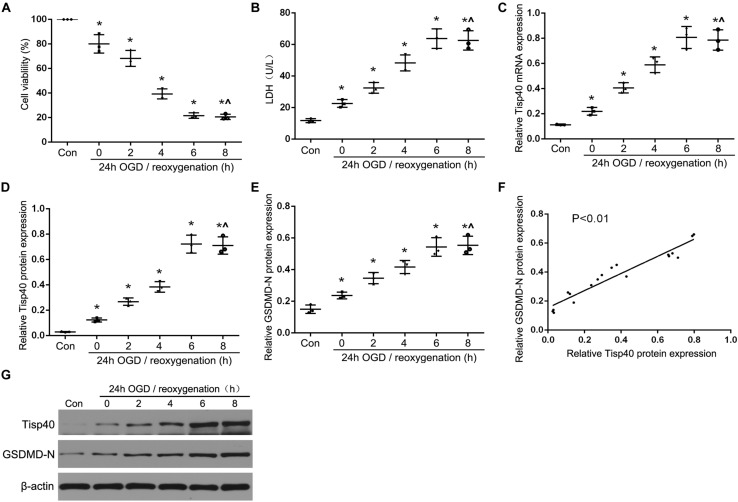
Tisp40 and GSDMD-mediated pyroptosis were induced in OGD/R-stimulated TCMK-1 cell. **(A)** Cell viability was detected by CCK-8 assay. **(B)** The LDH release was detected by activity assays. **(C,D,G)** qRT-PCR and western blot analysis of Tisp40 expression at different durations of reoxygenation after 24 h OGD in TCMK-1 cells; **(E,G)** Western blot analysis of GSDMD-N protein expression at different durations of reoxygenation after 24 h OGD in TCMK-1 cells. **(F)** The correlation between the protein expression of Tisp40 and GSDMD-N was analyzed by Pearson’s correlation analysis in TCMK-1 cells. Data are expressed as the mean ± SD. *n* = 5 per group. *P < 0.05 vs. con, ^∧^*P* > 0.05 vs. 24 h OGD and reoxygenation of 6 h, one-way ANOVA.

Our previous study showed that Tisp40 is upregulated in the kidney of I/R-induced mice (Xiao et al., 2017) and oxygen oxygen-glucose deprivation/reoxygenation (OGD/R)-induced TECs (Qin et al., 2017). To investigate if Tisp40 is involved in GSDMD-mediated TECs pyroptosis, samples were collected and assayed at 0, 6, 12, 24, or 48 h of reperfusion after 30 min of ischemia *in vivo*, and at 0, 2, 4, 6, and 8 h of reoxygenation after 24 h of OGD *in vitro*. As shown in Figure 1A, the loss of brush border detachment, interstitial congestion, and tubular cell death were aggravated, and TUNEL-positive TECs increased gradually with reperfusion time. At 30 min ischemia/24 h reperfusion, large numbers of renal tubules severely damaged, and most TECs were TUNEL-positive (Figures 1B,C). The expression of Tisp40 and GSDMD-N were evaluated in mice samples by western blotting, and Tisp40 was assessed using qRT-PCR. The results showed that Tisp40 and GSDMD-N expression increased gradually, peaked at 24 h, and was maintained until 48 h of reperfusion compared to the sham group (Figures 1D–F,H, *n* = 6 per group). As shown in Figure 2A, data from the CCK-8 assay demonstrated that OGD/R reduced the viability of TCMK-1 cells, which continuously decreased to approximately 40% at 6 h of reoxygenation and showing a significant difference compared to the Con group. OGD/R treatment stimulated significantly higher LDH release compared with the Con group; LDH release increased gradually over time, peaking at 6 h of reoxygenation in TCMK-1 cells (Figure 2B). Consistently in *in vivo* experiments, TCMK-1/wt cells treated with OGD/R also showed gradually elevated expression of Tisp40 and GSDMD-N, which peaked at 6 h and was maintained until 8 h of reperfusion compared to the Con group (Figures 2C–E,G, *n* = 3 per group). Furthermore, the data of pearson’s correlation analysis showed that the expression of Tisp40 and GSDMD-N was positively correlated in I/R-induced kidney and OGD/R-stimulated TECs (Figures 1G, 2F). These results indicate that Tisp40 is critical for GSDMD-mediated TEC pyroptosis.

### Tisp40 Enhanced Inflammasome Activation and Inflammatory Factors Release in Cultured OGD/R-Stimulated TCMK-1 Cells

**FIGURE 3:**
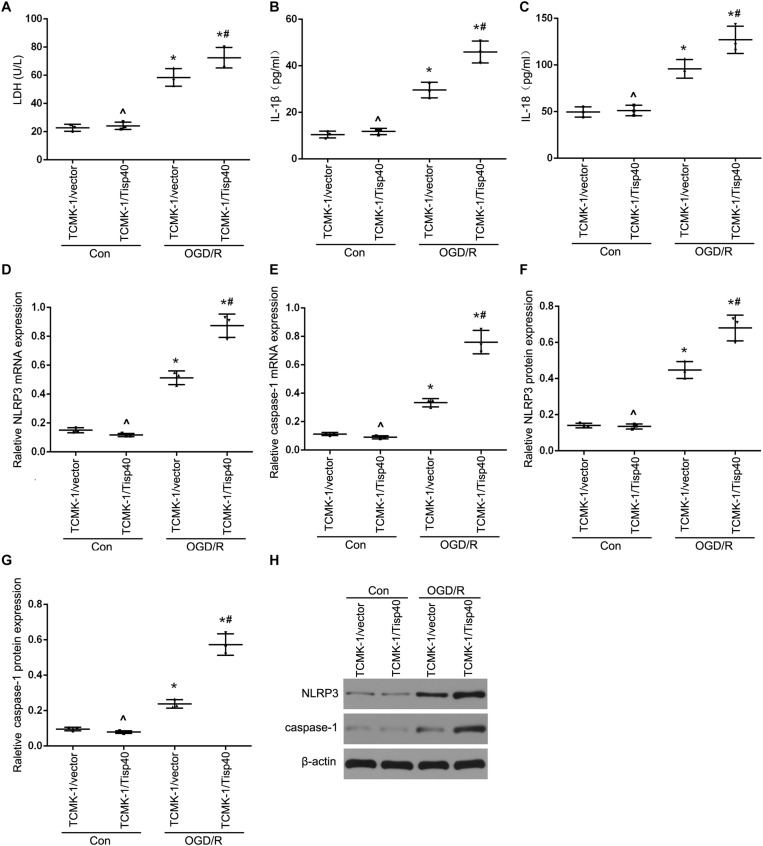
Tisp40 enhanced NLRP3 inflammasome activation in cultured OGD/R-stimulated TCMK-1 cells. **(A)** The LDH release was detected by activity assays. IL-1β **(B)** and IL-18 **(C)** contents were measured by using ELISA kits. **(D–H)** The expression of NLRP3 and caspase-1 were analyzed by qRT-PCR and western blot. Data are expressed as the mean ± SD. *n* = 5 per group. *P < 0.05 vs. corresponding control groups (Con). ^∧^*P* > 0.05 vs. TCMK-1/vector in con group. ^#^*P* < 0.05 vs. the group of TCMK-1/vector cells induced by OGD/R, one-way ANOVA.

**FIGURE 4:**
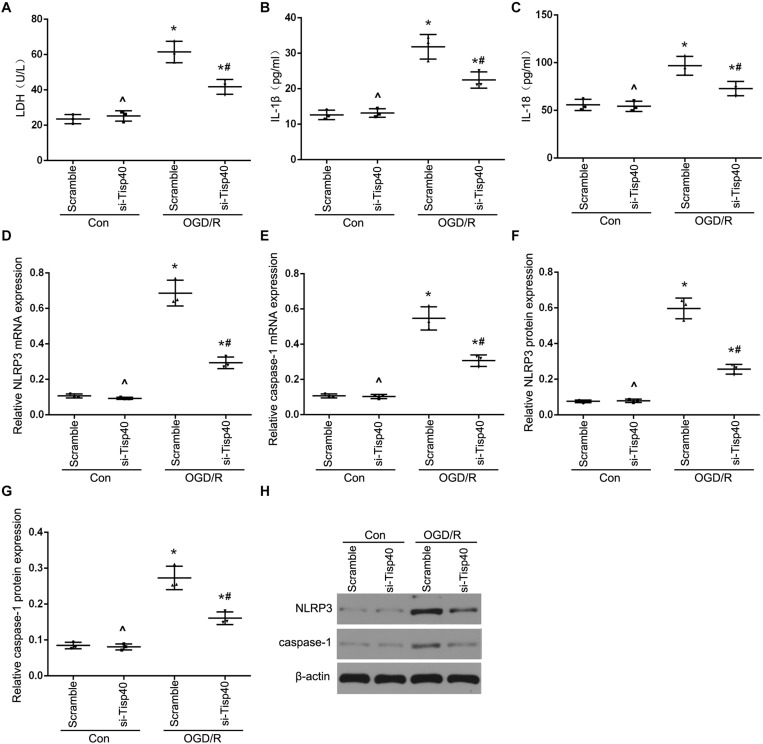
Tisp40 inhibitor alleviated NLRP3 inflammasome activation in cultured OGD/R-stimulated TCMK-1 cells. **(A)** The LDH release was detected by activity assays. IL-1β **(B)** and IL-18 **(C)** contents were measured by using ELISA kits. **(D–H)** The expression of NLRP3 and caspase-1 were analyzed by qRT-PCR and western blot. Data are expressed as the mean ± SD. *n* = 5 per group. *P < 0.05 vs. corresponding control groups (Con). ^∧^*P* > 0.05 vs. scramble con group. ^#^*P* < 0.05 vs. the group of Scramble induced by OGD/R, one-way ANOVA.

To further evaluate the functions of Tisp40 protein in pyroptosis, the effects of Tisp40 on NLRP3 inflammasome activation and IL-1β and IL-18 release were assessed in cultured OGD/R-stimulated TCMK-1 cells. A Tisp40-overexpressing cell line (TCMK-1/Tisp40) and si-Tisp40 were used to upregulate or downregulate Tisp40 *in vitro*, as described in our previous study (Xiao et al., 2017). Following treatment with OGD/R, LDH release increased significantly in cultured TCMK-1/Tisp40 cells compared to those in the control and empty vector groups (Figure 3A), but decreased significantly after Tisp40 silencing (Figure 4A). IL-1β and IL-18 were examined using ELISA, and NLRP3 and caspase-1 were measured using qPT-PCR and western blotting. Exposure of the cells to OGD/R resulted in an increase in the levels of IL-1β, IL-18, NLRP3, and caspase-1, which was aggravated by Tisp40 overexpression compared to that in the control and empty vector groups (Figures 3B–H), and relieved by Tisp40 silencing compared to that in the scramble group (Figures 4B–H). These results indicate that Tisp40 enhanced NLRP3 inflammasome activation and promoted the release of IL-1β and IL-18 in cultured OGD/R-stimulated TCMK-1 cells, supporting that Tisp40 is critical in pyroptosis of TECs.

### Tisp40 Knockout Protects Against Inflammasome Activation and Inflammatory Factors Release in I/R-Induced Kidney

**FIGURE 5:**
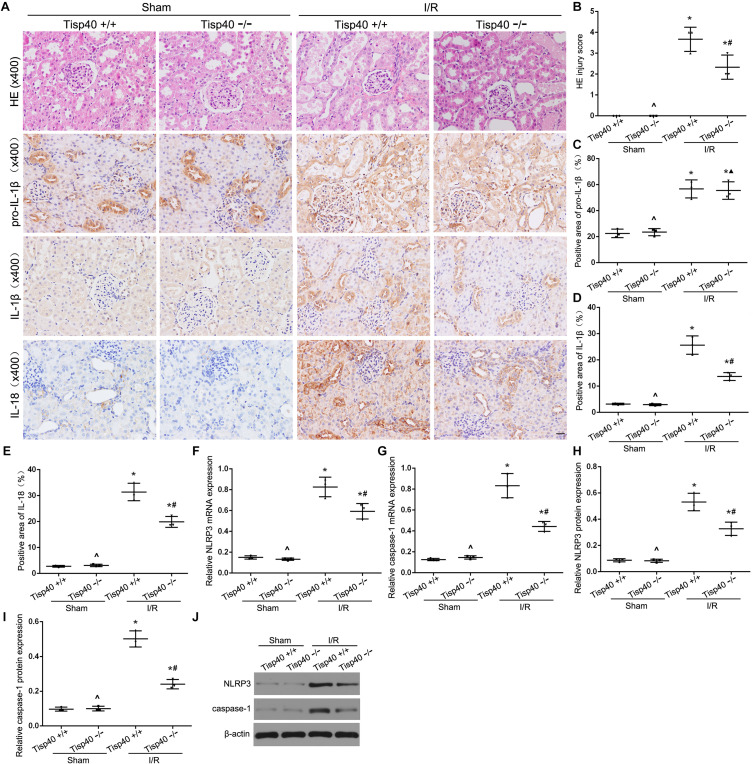
Tisp40 knockout protects against NLRP3 inflammasome activation in I/R-induced kidney. **(A)** Representative photomicrographs of tubular cell injury in mouse kidney tissue sections with HE staining and representative photomicrographs of pro- IL-1β, IL-1β, and IL-18 expression in mouse kidney tissue sections by immunohistochemistry, 400×, scale bar = 20 μm. **(B)** Statistical quantification analysis showed the injury score of HE staining in the kidney tissues **(C,D,E)** Statistical analysis showed the positive area of pro- IL-1β, IL-1β, and IL-18 in the kidney tissues. **(F–J)** The expression of NLRP3 and caspase-1 were analyzed by qRT-PCR and Western blot. Data are expressed as the mean ± SD. *n* = 6 per group. *P < 0.05 vs. corresponding sham-operation groups (Sham). ^∧^*P* > 0.05 vs. Tisp40^+/+^ in sham groups. ^#^*P* < 0.05, ^▲^*P* > 0.05 vs. the group of I/R-induced Tisp40^+/+^ mice. one-way ANOVA.

After determining the effects of Tisp40 on OGD/R-stimulated inflammasome activation and inflammatory factor release *in vitro*, we investigated the protective effect of Tisp40 deficiency on NLRP3 inflammasome activation and release of IL-1β and IL-18 using Tisp40-knockout (Tisp40^–/–^) and wild-type (Tisp40^+/+^) mice for *in vivo* analysis. We confirmed that Tisp40 was not expressed in the kidneys of Tisp40^–/–^ mice (Xiao et al., 2017). We compared renal pathologic changes in renal IRI of both genotypes induced by I/R. HE staining suggested that Tisp40 knockout could not change renal structures without the treatment of I/R. The kidneys of wild-type mice exhibited significantly elevated renal tubular damages, which in Tisp40-/- mice were markedly attenuated (Figures 5A,B). IHC staining revealed that the levels of pro-IL-1β, IL-1β, and IL-18 in kidneys were higher in I/R-induced mice than in sham-operation mice. There was no difference in pro-IL-1β in I/R-induced kidneys between Tisp40-/- mice and wild type mice, but both IL-1β and IL-18 were significantly inhibited in I/R-induced kidneys of Tisp40-/- mice. (Figures 5A,C–E). Furthermore, the qRT-PCR and western blotting results showed that NLRP3 and caspase-1 were significantly inhibited in the kidneys of Tisp40^–/–^ mice induced by I/R (Figures 5F–J).

### Tisp40 Is an Essential Regulator of GSDMD-Mediated Pyroptosis in TECs

**FIGURE 6:**
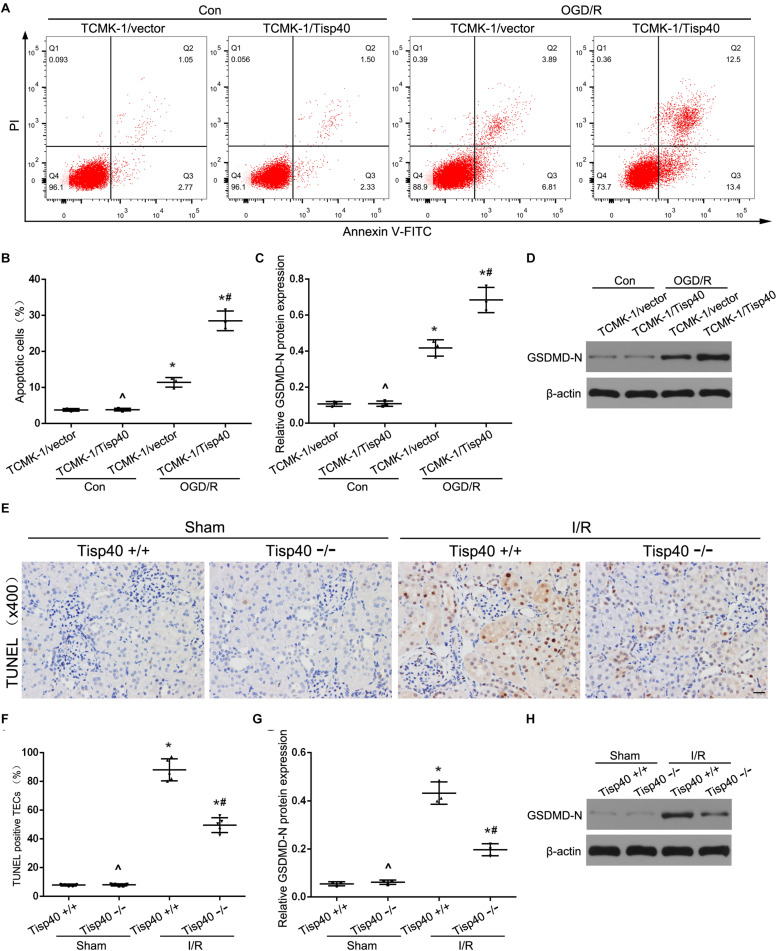
Tisp40 was an essential regulator of GSDMD-mediated TECs pyroptosis. **(A)** Flow cytometry assays were performed to show the cell pyroptosis. **(B)** Statistical analysis was used to show pyroptosis cells. **(C,D)** The expression of GSDMD-N in TCMK-1 cells were analyzed by Western blot. **(E)** Representative photomicrographs of tubular cell injury in mouse kidney tissue sections with TUNEL staining, 400×, scale bar = 20 μm. **(F)** Statistical analysis showed the percentage of TUNEL positive TECs in the kidney tissues exposed to sham or I/R. **(G,H)** The expression of GSDMD-N in kidneys were analyzed by Western blot. Data are expressed as the mean ± SD. *n* = 5 per group *in vitro* and *n* = 6 per group *in vivo*. ^P>0.05 vs. TCMK-1/vector cells in con group or. Tisp40^+/+^ mice in sham group. *P < 0.05 vs. corresponding control groups (Con) or sham-operation groups (Sham). ^#^*P*< 0.05 vs. TCMK-1/vector cells in OGD/R group or Tisp40^+/+^ I/R group, one-way ANOVA.

We next investigated the effect of Tisp40 on GSDMD-mediated pyroptosis in TECs. Flow cytometry data showed that TCMK-1 cell pyroptosis was induced by 24 h of OGD/6 h of reoxygenation and that Tisp40 overexpression markedly promoted cell pyroptosis in TCMK-1/Tisp40 compared to those in the control and empty vector groups (Figures 6A,B). The level of GSDMD-N was examined using western blotting, which showed that the expression of OGD/R-stimulated GSDMD-N in TCMK-1/Tisp40 cells was markedly higher than those in the control and empty vector groups (Figures 6C,D). The TUNEL assay revealed no obvious pyroptosis of TECs in the sham group. Compared to the Tisp40^+/+^ group, Tisp40 knockout obviously decreased the number of TUNEL-positive TECs induced by I/R (Figures 6E,F). In addition, GSDMD-N was significantly inhibited in the I/R-induced kidneys of Tisp40-knockout mice compared to that in the wild-type mice (Figures 6G,H). Based on the role of Tisp40 in regulating inflammasome activation and inflammatory factors release in I/R induced mice and OGD/R stimulated TCMK-1 cells, it is further illustrated that Tisp40 is an essential regulator of GSDMD-mediated TEC pyroptosis.

### Tisp40 Regulated GSDMD-Mediated TECs Pyroptosis via NF-κB Pathway

**FIGURE 7:**
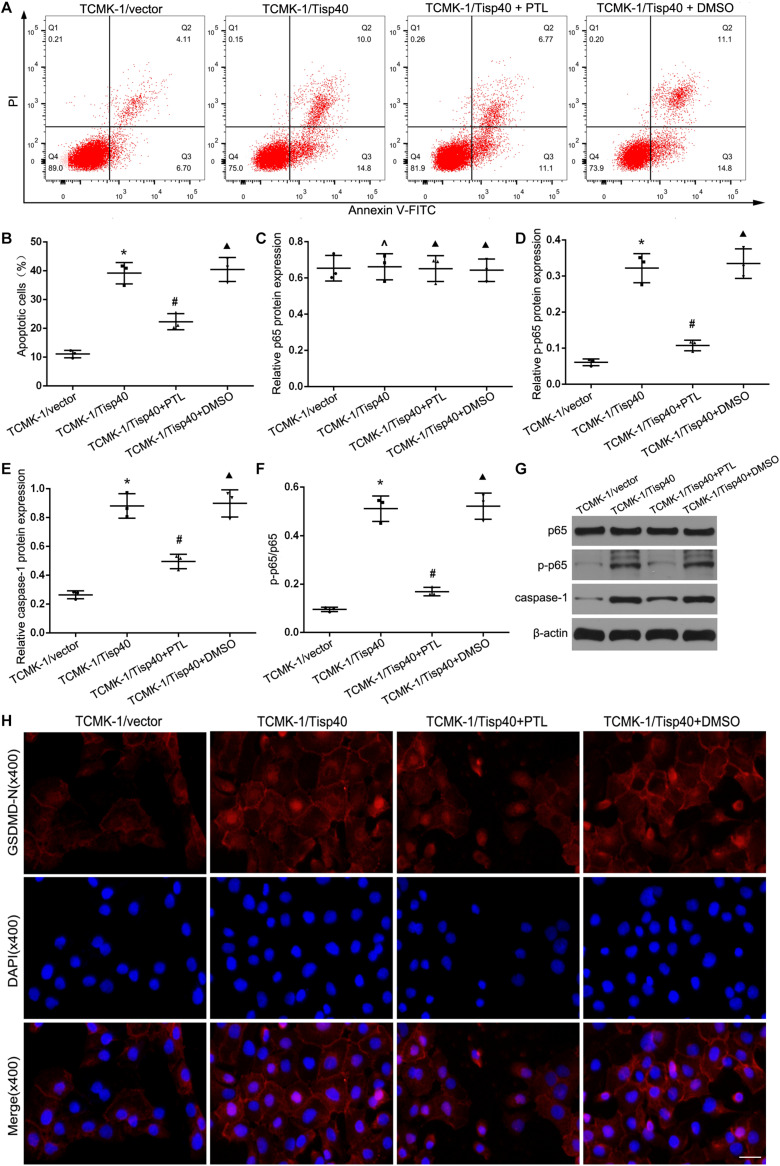
Tisp40 regulated GSDMD-mediated pyroptosis via NF-κB pathway in OGD/R stimulated TCMK-1 cell. **(A)** Flow cytometry assays were performed to show the cell pyroptosis. **(B)** Statistical analysis was used to show pyroptosis cells. **(C,D,E,G)** The expression of p65,p-p65 and caspase1 were analyzed by Western blot. **(F)** The ratio of p-p65 to p65 reflect phosphorylation level of p65. **(H)** Representative photomicrographs of GSDMD-N expression in TCMK-1 cells by Immunofluorescence, 400×, scale bar = 20 μm. Data are expressed as the mean ± SD. *n* = 5 per group. *P < 0.05, ^∧^*P* > 0.05 vs. TCMK-1/vector group. ^#^*P* < 0.05, ^▲^*P* > 0.05 vs. TCMK-1/Tisp40 group, one-way ANOVA.

Studies have shown that the NF-κB signaling pathway can activate GSDMD-related pyroptosis in tubular cells (Wang et al., 2019). As shown in Figures 7A,B, we found parthenolide (PTL, NF-κB inhibitor) alleviated cell pyroptosis in the TCMK-1/Tisp40 group. To further evaluate the signal transduction pathways involved, we confirmed that the levels of p-p65 and caspase-1 were higher in the OGD/R-induced TCMK-1/Tisp40 group than in the control group. However, the effect of Tisp40 overexpression on p-p65 and caspase-1 abundance was partially blocked by PTL (Figures 7C–G). Additionally, PTL reduced OGD/R-induced GSDMD-N expression in TCMK-1/Tisp40 cells according to immunofluorescence analysis. These results indicate that Tisp40 regulates GSDMD-mediated pyroptosis in TECs via the NF-κB pathway.

## Discussion

Renal IRI is a common cause of AKI and easily develops into CKD and ESRD, creating societal and personal economic burdens (Tao et al., 2013; Chawla et al., 2014). No effective drugs or related treatment strategies are available except for renal replacement therapy to prolong survival (Kaushal and Shah, 2014). Determining the underlying mechanism of renal IRI is important for developing treatments for AKI (Agarwal et al., 2016). Various mechanisms are involved in renal IRI, including oxygen radicals, intracellular calcium overload, inflammatory response, and apoptosis (Linkermann et al., 2014); however, the mechanism of pyroptosis in renal IR is poorly understood. We examined the function of the Tisp40 gene and its regulatory relationship in the pyroptosis of renal IRI. We found that Tisp40 was closely related to GSDMD-mediated pyroptosis in TECs, and Tisp40 deficiency inhibited GSDMD-mediated pyroptosis by inhibiting NLRP3, caspase1, IL-1β, and IL-18 via the NF-κB pathway in I/R-induced kidneys and OGD/R-induced TECs.

We and others reported that Tisp40 was a regulator in injury of TECs induced by I/R, and Tisp40 knockout effectively alleviated I/R-induced renal fibrosis, apoptosis and inflammation (Qin et al., 2017, 2018; Xiao et al., 2017). Under physiological conditions, Tisp40 is mainly located on the endoplasmic reticulum. When stimulated by external stimuli, Tisp40 is transferred by Golgi body site 1 protease to the nucleus to regulate downstream target genes (Nagamori et al., 2005). In concurrence with the development of renal tubular injury, Tisp40 expression was gradually elevated and peaked at 30 min ischemia/24 h reperfusion. This not only illustrated that renal IRI model was established successfully, also showed Tisp40 gene expression could be an important molecular event in renal IRI and play a role in regulating biological behavior of TECs. Further results showed the exposure to OGD/R led to an abundance of Tisp40 in TEC.

Pyroptosis, characterized by excessive cell death and inflammation, is a specific cell death program distinct from apoptosis and necrosis (Miao et al., 2010). In this study, we found the injury induced by I/R and OGD/R resulted in excessive TEC death, which always went with pyroptosis in renal IRI (Tajima et al., 2019; Wang et al., 2019; Liu et al., 2020). As the main executor of cell pyroptosis, GSDMD, is widely expressed in different cells and tissues (Shi et al., 2015; Liu et al., 2016). During pyroptosis, the N-terminal domain of GSDMD (GSDMD-N) and cell membrane combine, leading to the formation of cell membrane pores, which causes the cells to swell and disrupt, releasing inflammatory factors, such as LDH (Byrne et al., 2013; Shi et al., 2015; Whitfield et al., 2017). In our study, GSDMD-N expression increased gradually with injury of TECs. Simultaneously, the level of LDH release was correspondingly increased in cultured TCMK-1 cells. We suggest that TEC pyroptosis appeared at early stage of renal IRI. In addition, the levels of GSDMD-N expression and LDH release peaked at 30 min ischemia/24 h reperfusion in *in vivo* and 24 h OGD/R/6 h reoxygenation *in vitro*. These results indicated TECs pyroptosis was an efficient and speedy cell death program. We also provided a method for model establishment of TEC pyroptosis in renal IRI. Besides, the expressions of Tisp40 and GSDMD-N were positively correlated, which revealed Tisp40 might be involved in GSDMD-mediated TEC pyroptosis.

Pyroptosis, marked by inflammasome formation and requires caspase-1 activation, is an adaptive immune in response to intra-and extracellular stimuli (Shi et al., 2016). As an inflammatory molecule, NLRP3 (nod-like receptor protein-3) is activated by binding to the precursor of caspase-1 (procaspase-1) and adapter protein ASC, after which procaspase-1 is converted into catalytic caspase-1, which promotes the maturation and release of IL-1β and IL-18, triggering a strong inflammatory response (Miao et al., 2010; Xi et al., 2016). Notably, we observed the characteristic hallmarks of pyroptosis in renal IRI, including increased activation of NLRP3 inflammasome and caspase-1, and the release of IL-1β and IL-18. We further explored the mechanism of Tisp40’s involvement in TEC pyroptosis. We found Tisp40 overexpression aggravated the expressions of NLRP3 and caspase-1, and the release of LDH, IL-1β and IL-18, whereas the opposite occurred in TECs treated with siRNA targeting Tisp40. These findings was further confirmed *in vivo*. Interesting, we found that there was no change in the expression of pro-IL-1β between Tisp40-deficient mice and wild-type mice, which suggests that the protective effect of Tisp40 deficiency might suppress the inflammatory factors mature, rather than inhibit the expressions of inflammatory cytokines precursors. Subsequently, the nature of cell death was monitored by flow-cytometry analyze *in vitro* and TUNEL staining *in vivo*. Tisp40 overexpression markedly promoted pyroptosis in TECs induced by OGD/R, and Tisp40 knockout alleviated TEC pyroptosis in I/R-induced kidney. Furthermore, the expression of GSDMD-N was detected to verify the role of Tisp40 in pyroptosis. We found that Tisp40 overexpression markedly promoted the expression of GSDMD-N in TECs induced by OGD/R, and Tisp40 knockout alleviated the expression of GSDMD-N in I/R-induced kidney. Collectively, it is confirmed that Tisp40 has transcriptional regulation function on GSDMD-midiated pyroptosis in renal IRI.

As a nuclear transcription factor commonly present in the cytoplasm in the form of a homo- or hetero dimer, NF-κB is a key regulator of TEC pyroptosis by activating the NLRP3 inflammasome and promoting proinflammatory responses (Gordon et al., 2011; Yang et al., 2014). In addition, NF-κB activated GSDMD-related pyroptosis in tubular cells (Wang et al., 2019). Moreover, Tisp40 deficiency limits renal inflammation by inhibition of phosphorylation NF-κB (Qin et al., 2018). Therefore, we predicted that the NF-κB was a bridge between Tisp40 and TEC pyroptosis. As expected, Tisp40 overexpression significantly increased the expression of p-p65 in OGD/R-stimulated cells, while the effects of Tisp40 overexpression on GSDMD-mediated pyroptosis were partially blocked by parthenolide (an inhibitor of NF-κB). These results demonstrate that Tisp40 regulate GSDMD-mediated TECs pyroptosis via phosphorylation of p65.

## Conclusion

**FIGURE 8:**
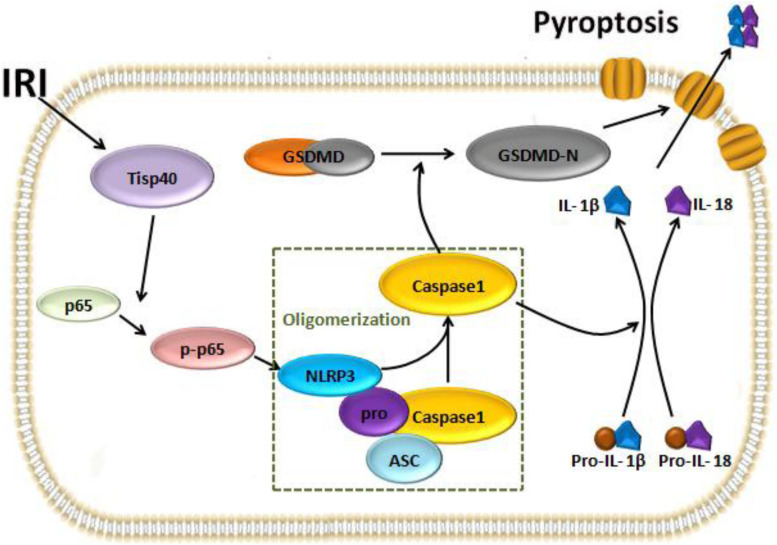
The pathway that Tisp40 regulates Ischemia-reperfusion induced pyroptosis in kidney.

In conclusion, our study indicates Tisp40 is an essential regulator of GSDMD-mediated pyroptosis in TECs via nuclear factor-κB p65 activation, and Tisp40 inhibition protects against tubular epithelial cell GSDMD-mediated pyroptosis in renal ischemia-reperfusion injury (Figure 8). Combined with our previous research results, this study further confirms that Tisp40 may be a promising therapeutic target for preventing AKI and its evolution to CKD and ESRD.

## Data Availability Statement

The raw data supporting the conclusions of this article will be made available by the authors, without undue reservation.

## Ethics Statement

The animal study was reviewed and approved by the Institutional Animal Care and Use Committee of Qingdao Municipal Hospital.

## Author Contributions

CX and RW designed the research, analyzed the data, and drafted the manuscript. HZo and QS performed the experiments. CX, HZu, and YZ helped with data acquisition and discussion. CX and RW analyzed the data and prepared the figures. All authors contributed to manuscript writing and editing.

## Conflict of Interest

The authors declare that the research was conducted in the absence of any commercial or financial relationships that could be construed as a potential conflict of interest.
